# Sexual Dimorphism in the Human Olfactory Bulb: Females Have More Neurons and Glial Cells than Males

**DOI:** 10.1371/journal.pone.0111733

**Published:** 2014-11-05

**Authors:** Ana V. Oliveira-Pinto, Raquel M. Santos, Renan A. Coutinho, Lays M. Oliveira, Gláucia B. Santos, Ana T. L. Alho, Renata E. P. Leite, José M. Farfel, Claudia K. Suemoto, Lea T. Grinberg, Carlos A. Pasqualucci, Wilson Jacob-Filho, Roberto Lent

**Affiliations:** 1 Institute of Biomedical Sciences, Federal University of Rio de Janeiro, Rio de Janeiro, Brazil; 2 Aging Brain Study Group, University of São Paulo Medical School, São Paulo, Brazil; 3 Department of Neurology and Pathology, University of California San Francisco, San Francisco, United States of America; 4 National Institute of Translational Neuroscience, Ministry of Science and Technology, São Paulo, Brazil; 5 Discipline of Geriatrics, University of São Paulo Medical School, São Paulo, Brazil; 6 Brain Institute, Hospital Israelita Albert Einstein, São Paulo, Brazil; Université Lyon, France

## Abstract

Sex differences in the human olfactory function reportedly exist for olfactory sensitivity, odorant identification and memory, and tasks in which odors are rated based on psychological features such as familiarity, intensity, pleasantness, and others. Which might be the neural bases for these behavioral differences? The number of cells in olfactory regions, and especially the number of neurons, may represent a more accurate indicator of the neural machinery than volume or weight, but besides gross volume measures of the human olfactory bulb, no systematic study of sex differences in the absolute number of cells has yet been undertaken. In this work, we investigate a possible sexual dimorphism in the olfactory bulb, by quantifying postmortem material from 7 men and 11 women (ages 55–94 years) with the isotropic fractionator, an unbiased and accurate method to estimate absolute cell numbers in brain regions. Female bulbs weighed 0.132 g in average, while male bulbs weighed 0.137 g, a non-significant difference; however, the total number of cells was 16.2 million in females, and 9.2 million in males, a significant difference of 43.2%. The number of neurons in females reached 6.9 million, being no more than 3.5 million in males, a difference of 49.3%. The number of non-neuronal cells also proved higher in women than in men: 9.3 million and 5.7 million, respectively, a significant difference of 38.7%. The same differences remained when corrected for mass. Results demonstrate a sex-related difference in the absolute number of total, neuronal and non-neuronal cells, favoring women by 40–50%. It is conceivable that these differences in quantitative cellularity may have functional impact, albeit difficult to infer how exactly this would be, without knowing the specific circuits cells make. However, the reported advantage of women as compared to men may stimulate future work on sex dimorphism of synaptic microcircuitry in the olfactory bulb.

## Introduction

Aside from obvious bodily differences, men and women reportedly differ in their cognitive characteristics, despite a great controversy on the role of biological versus social determinants on each of these sex differences [1], [2]. For many of them, biological correlates have been reported, such as anatomical characteristics of brain areas related to language [3], electrophysiological surrogates of emotion [4], hormonal influences on behavior [5], and functional neuroimaging activation after cognitive tasks such as attention and memory [6].

Sex differences on olfactory detection, perception, and their cognitive implications have also been reported, all considered to play an important role on differentiated social behaviors of men and women [7], [8]. The same “nature vs nurture” controversy is prevalent for this sensory modality. Despite social and regional differences in attitudes towards olfaction, however, women usually show a higher interest in the sense of smell than men [9], and perform better in many specific tasks involving olfaction [10]–[20].

Examples for the relevance of sexual dimorphism in olfaction are the changing olfactory sensitivity of women during the menstrual cycle [10], its importance in behavior towards sexual partners [11], and its influence on social communication [7], [12]. In addition, mutual recognition of mothers and their babies by odor [13], and of women and men by olfactory, possibly pheromonal cues [14], represent other instances of the biological and behavioral importance of these sex differences.

Males and females differ on the perceptual evaluation of odor intensity, as shown, pioneeringly, by Doty [15], who observed that only adult women rated as strong or extremely strong the exposure to some particular scents such as crystalline exaltolide, a result that was not maintained neither for postmenopausal women, nor for prepubescent girls, suggesting that the higher response was related to ovarian hormones. In more complex tasks, as the suprathreshold perception of odorants associated to the attribution of emotional valence, women reported feeling more pleasure to camphor, menthol, citronella and ferric valerian than men, while the latter preferred cedar wood oil, pine oil, musk, and tonka beans [10]. Similarly, several other studies by the same group revealed sex differences in intensity and pleasantness of body odors: for example, women show higher intensity and lower pleasantness to vaginal [16], axillary [17] and breath odors than men [18]. In another study [19], the intensity, pleasantness, irritation, familiarity, and coolness of 50 microencapsulated odors were quantified through visual analogue scales by women and men of different ages (5–99 years old), and rated more intense, less cool, less irritating, more familiar and more unpleasant by women as compared with men, independently of age. Other groups have arrived at similar conclusions with different experiments [20], [21]. Based on these multiple data, it can safely be concluded not only that olfaction is a sex-specific function, but also that women outperform men in different aspects of olfactory sensitivity [10], [22], irrespective of their age.

To distinguish between socially driven sex differences, and biologically determined ones, it is important to investigate and find the neural correlates of behavioral/cognitive specificities of each sex. Some attempts have been made, and evidence has been produced by electrophysiological recording of event-related potentials [23]–[25], confirming the superiority of women in odor detection and emotional valence. Functional neuroimaging, on the other hand, has proved controversial so far, with negative [26] and positive [27] evidence for olfactory dimorphism, the latter favoring superior olfactory abilities by women. Morphological measures of olfactory regions, as well, provided discrepant evidence: olfactory bulb volumes were found to be greater in adult men as compared with women [28], but not in children and adolescents [29]. Other olfactory regions showed to be larger either in males, or in females [30].

Neuroimaging methods, however, only reveal indirect, gross measures of brain structures. The absolute number of cells, for this purpose, may be a more accurate parameter, disclosing whether the processing units in the brains of women and men do differ. A larger number of neurons in one sex, for instance, would tell us that the processing machinery of the region investigated would be prepared to perform better in functional terms, although the actual mechanisms of this differentiated perception would depend on data about synaptic circuits in each region.

We sought to approach this issue by investigating sexual dimorphism in the human olfactory bulb, the first processing locus of the olfactory chain after peripheral transduction of odors. The olfactory bulb is believed to host the first stages of processing olfactory stimuli, since it is the locus where primary axons from peripheral receptor neurons interact with a more complex functional network at the glomeruli [31]. Also, synaptology of their cellular constituents is different from other regions, greatly involving dendrodendritic, reciprocal contacts of granular interneurons [32], the most numerous neuronal type in this region. Such a distributed, axonless, nonpolarized architecture would potentially depend on the number of units [33], an important reason why investigating OB cellularity in humans achieves a particular relevance. In addition, unlike other mammals [34], the adult human olfactory bulb has recently been shown to lack neurogenesis [35], despite some indirect evidence for its existence [36].

We thus investigated quantitatively the cellularity of the OB by taking advantage of a recently developed technique that allows determination of absolute cell composition in the brain - the isotropic fractionator [37]. Our hypothesis was that, since women outperform men in many olfactory functions, they would show a larger number of cells in the olfactory bulb, as compared with men.

## Materials and Methods

### Subjects

The study was approved by the Ethics Committees of both the University of São Paulo (Comitê de Ética em Pesquisa, Proc. 337/10) and of the Federal University of Rio de Janeiro (Comitê de Ética em Pesquisa, Proc. 588/10). Donation of the brains was authorized by primary caretakers through written, informed consent, as well as the scales applied to them, to assure cognitive normality of the subjects.

**Figure 1:**
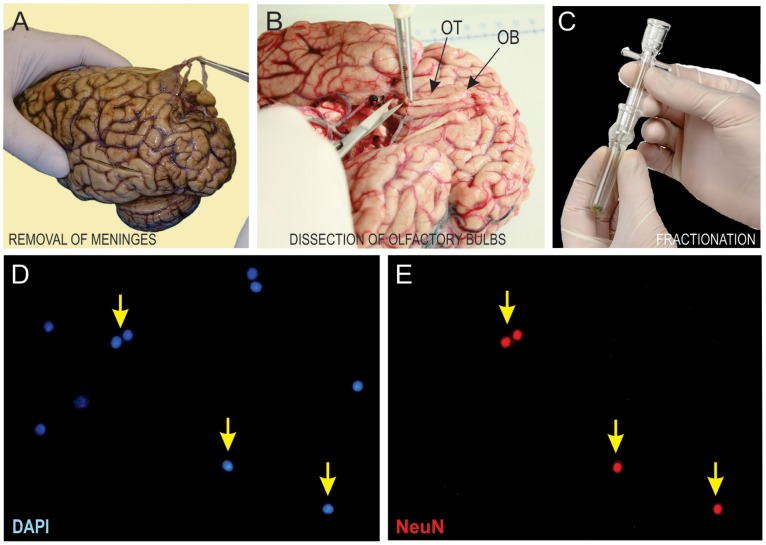
Stages and instruments of the methodological procedures. In **A**, removal of meninges and blood vessels. **B**, dissection of the right olfactory bulb (OB) and tract (OT). **C**, fractionation in a tissue homogenizer. **D**, DAPI-stained nuclei in the Neubauer chamber. **E**, NeuN-positive nuclei. The yellow arrows show nuclei that colocalize DAPI and NeuN, therefore considered to be neuronal, and counted as such.

Eighteen olfactory bulbs (Table 1), seven from male (58–92 years old) and eleven from female (55–94 years old) Brazilian subjects, were obtained 8 to 18 hours after death from the Brain Bank of the Brazilian Aging Brain Study Group [38] of the University of São Paulo Medical School. All subjects resided in the same environment (the city of São Paulo, Brazil) under moderate to high levels of pollution, and had daily activities not particularly dependent on olfactory functions (no professional cooks, coffee-tasters, etc). The corpses were kept in a cold room at 4°C soon after death until the time of autopsy. After that period, the brains were removed from the cranium, meninges were dissected out (Figure 1A), and the olfactory bulbs and tracts were separated (Figure 1B), placed in cassettes and fixed by immersion in 2% paraformaldehyde (PFA) for a period of 36 to 40 hours. They were maintained in PBS at 4°C until the day of chemomechanical dissociation.

**Table 1:** Characteristics of the sample.

Case	Sex	Age (years)	CDR	IQCODE	Braak	CERAD	APD*	OB mass, g (left or right)	Cause of death
**1**	M	58	0	3	1	0	Normal	0.109 (R)	Pulmonary edema
**2**	M	75	0	3	0	0	Normal	0.124 (L)	Pulmonary edema
**3**	M	76	0	3	1	0	Normal	0.143 (L)	Pulmonary edema
**4**	M	77	0	3	2	0	AGD	0.147 (R)	Pulmonary edema
**5**	M	85	0	3	2	A	Normal	0.137 (L)	Acute anemia
**6**	M	86	0	3	2	A	AGD	0.154 (L)	Hemopericardium
**7**	M	92	0	3	2	0	Normal	0.144 (L)	Acute pulmonar edema
**8**	F	55	0	3	2	0	Normal	0.106 (L)	Pulmonary edema
**9**	F	56	0	3	1	0	Normal	0.129 (R)	Pulmonary edema
**10**	F	59	0	3	1	A	NA	0.128 (R)	Pulmonary embolism
**11**	F	61	0	3	1	0	Normal	0.128 (R)	Pulmonary shock
**12**	F	65	0	3	1	Moderate	Normal	0.118 (R)	Hipovolemic shock
**13**	F	76	0	3	NA	0	AGD	0.242 (L)	Pulmonary thromboembolism
**14**	F	77	0	3	3	B	Normal	0.132 (L)	Pulmonary shock
**15**	F	79	0	3	3	C	ASYMAD	0.131 (R)	Pulmonary embolism
**16**	F	80	0	3	4	B	ASYMAD	0.118 (L)	Pulmonary edema
**17**	F	84	0	3	NA	0	AGD	0.115 (L)	Hemoperitoneum
**18**	F	94	0	3,11	3	A	AGD	0.108 (R)	Pulmonary edema

**Abbreviations**: AGD = argyrophilic grain disease; APD = anatomopathological diagnosis; ASYMAD = asymptomatic Alzheimer’s disease; F = female; M = male; NA = not available.

*Morphological markers for Alzheimer’s and argyrophilic grain disease were found in some cases, but were maintained in the sample since changes in cell numbers are reportedly related to dementia, rather than to plaques and tangles [42].

The clinical and functional status of the subjects was evaluated through a semi-structured interview applied to reliable caregivers. The questionnaires were applied by skilled nurses and included the Clinical Dementia Rating Scale (CDR) [39] and the Informant Questionnaire on Cognitive Decline in the Elderly (IQCODE) [40]. Only cognitively normal subjects (CDR = 0 and IQCODE = 3.0) with no or irrelevant pathological markers in their brains were included in this study. Cases with neurological and psychiatric disorders were excluded. Information on olfactory abilities of the subjects was not possible to obtain reliably from caretakers.

Information regarding age, sex, clinical history and cause of death is shown in Table 1. Brazilian subjects can be considered representative of many different ethnic ancestries, as revealed by genomic DNA analysis [41], what ascribes a general value to the results here reported. The mean age of subjects was 71.5 years (range 55–94) for females and 78.4 years (range 58–92) for men. No significant difference in age was found between males and females (unpaired t test, p = 0.255). The olfactory bulbs were divided into two groups: female (F) and male (M), both with age superior to 55 years old.

### Experimental procedures

A suspension of nuclei was obtained through mechanical dissociation of each fixed olfactory bulb in a standard solution (40 mM sodium citrate and 1% Triton X-100), using a 7 ml glass Tennbroeck tissue homogenizer (Figure 1C). Complete homogenization was achieved when the smallest visible fragments were dissolved. This technique – the isotropic fractionator - was described previously by Herculano-Houzel and Lent [37], applied successfully to human [42], [43] and animal [44], [45] brains, and validated in comparison with other quantitative techniques [46]. The isotropic fractionator applied to fixed tissue causes plasma membrane lysis with no damage to the nuclei, transforming the highly anisotropic brain tissue into a isotropic suspension of cell nuclei, which can then be stained and counted.

The olfactory bulbs homogenates were collected with a Pasteur pipette and transferred to flasks. To avoid loss of nuclei, the grinding pestle and mortar were washed several times with the dissociation solution. For quantifying all cell nuclei, 4,6-diamidino-2-phenylindole dihydrochloride (DAPI), a fluorescent DNA-specific dye (Molecular Probes, Eugene, OR) was added to the suspension. After sufficient agitation, aliquots of 10 µl were collected and deposited into a hemocytometer (Neubauer chamber). DAPI-stained nuclei (Figure 1D) were then counted using a fluorescence microscope. The suspension was considered homogeneous when counts varied by less than 10% across aliquots. Once nuclear density in the suspension was determined by averaging eight samples, the total number of cells was estimated by multiplying the mean nuclear density by the total suspension volume.

For estimates of total neuron number, a 1 ml aliquot was removed from the nuclear suspension and immunoreacted for NeuN, a neuron-specific nuclear marker [47]. It is important to stress that nuclear markers for absolute cell counting have to be specific (exclusive for a single cell type) and universal (positive for all cells of a given type). This is true for NeuN, which fails to stain only a few, low number neurons such as OB mitral cells, cerebellar Purkinje cells, retinal photoreceptors and some brainstem neurons [47]. The quantitative impact of these neurons, however, is irrelevant as compared with the total numbers achieved by absolute counting.

Nuclei in the aliquot were collected by centrifugation and resuspended in PBS. Subsequently, nuclei were again collected by centrifugation, washed in PBS, and incubated overnight at room temperature with anti-NeuN mouse IgG (1∶200 in PBS; Chemicon, USA). Mitral cell nuclei are reportedly not immunoreactive to anti-NeuN antibody [47]–[49]. However, the proportion of such cells in the human olfactory bulb is small compared to the total cell numbers [50], estimated to be only 50,935 in average at age 25; 32,718 at age 60; and 14,501 at age 95, figures that represent about 0.2–0.5% of total neuron numbers in the OB.

After washing in PBS, the nuclei were incubated in the secondary antibody Alexa Fluor 555 anti-mouse goat IgG (Figure 1E) (Molecular Probes, USA, 1∶150 in PBS, 10% goat serum, and 40% DAPI) for 2 h, collected by centrifugation, washed in PBS, and then resuspended in a small volume of PBS for counting under the fluorescence microscope.

The absolute cell numbers were calculated by multiplying the average nuclear density obtained in the isotropic aliquots stained with DAPI, by the total volume of the suspension. The neuronal cell number was obtained by counting the number of NeuN+ nuclei among about 500 nuclei stained with DAPI in each central fields of the hemocytometer chamber. The total number of non-neuronal nuclei was derived by subtraction of the number of NeuN+ nuclei from the total number of nuclei.

### Statistical analysis

Variables were described as mean **±** standard deviation (SD). Normal distribution was assessed using the Anderson-Darling test, and unpaired t-tests were used to compare OB weights and number of cells and neurons between sex groups, since the distribution proved to be normal. Because the ratio between non-neuronal over neuronal numbers for females were not normally distributed, the Mann-Whitney test was used in this case. The same was done for the male group, since the number of cases did not allow proper application of the Anderson-Darling test. Statistical analysis was performed with the Graph Prism 5.0 software. All tests had the significance level set at 0.05.

## Results

We determined the absolute cell number in eighteen human olfactory bulbs (either right or left) employing the isotropic fractionator method. The mean olfactory bulb mass was 0.132 g in females and 0.137 g in men (Table 1). No statistical difference was found for age (p = 0.255) and olfactory bulb mass (p = 0.765) between the groups. No significant differences were found, as well, for mass, neuronal, non-neuronal, or total cell numbers between right and left olfactory bulbs of both groups (data not shown). The values described below, therefore, refer to one bulb, disregarding side.

### Absolute Cell Number

**Figure 2:**
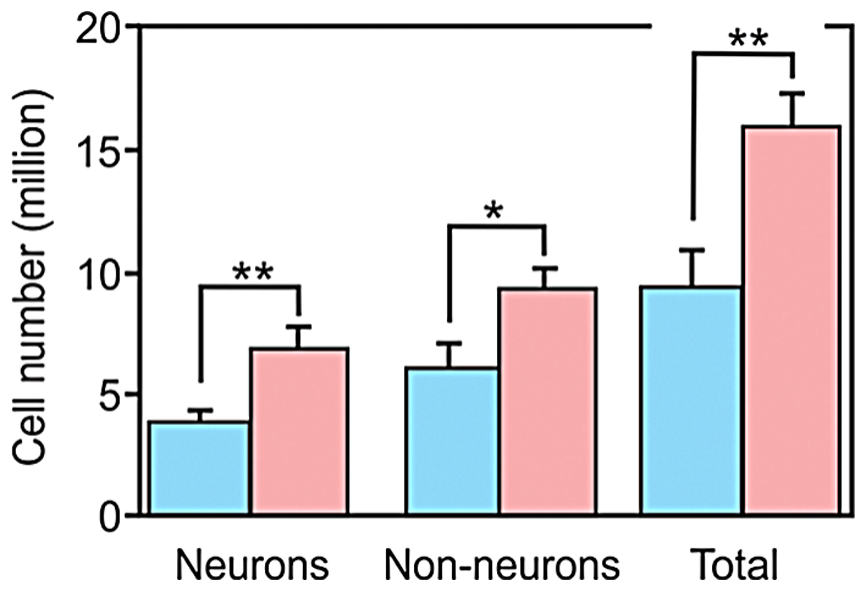
Average number of cells in the olfactory bulb of men and women. Blue bars indicate the mean number of cells in males, pink bars for females. Significant differences were found for the total number of cells, as well as for the number of neurons and non-neuronal cells. In all cases, women outnumbered men. Error bars indicate standard deviation. * p<0.05; ** p<0.01.

Women showed higher absolute number of cells than men: 16.2 million, against 9.2 million in males, a significant difference of 43.2% (p = 0.005) (Figure 2).

### Absolute Neuronal Number

The number of neurons in females reached 6.9 million, being no more than 3.5 million in males, a difference of 49.3% (p = 0.007, Figure 2).

### Absolute Non-neuronal Number

A significant difference of 38.7% was found also for non-neuronal cell number between males and females (p = 0.020, Figure 2). Non-neuronal number in males was 5.7 million and in females it was 9.3 million.

### Density

**Figure 3:**
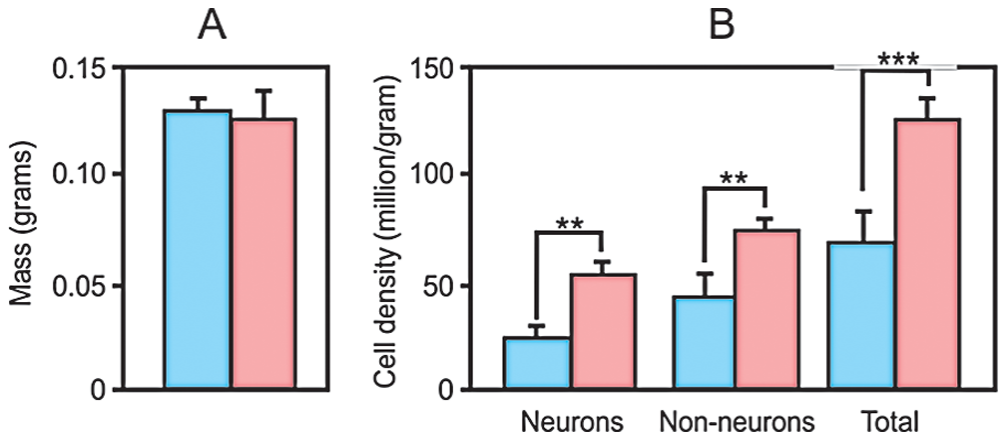
Olfactory bulb mass and cell density in men and women. In **A**, mean mass of olfactory bulbs for each sex. No difference was found between males and females. In **B**, mean neuronal, non-neuronal and total density in men and women. Blue bars represent males, pink bars for females. Error bars indicate standard deviation. **p<0.01; ***p<0.005.

Results remained significant when corrected for mass, i.e., when the density of cells was estimated for females and males. Women had a higher density of neurons, non-neurons and total cells than men. Total density was 125.9 million/g in women and 66.6 million/g in men (p = 0.002), a significant difference of 47.1%. Neuronal density was 48.4% higher in females than in males (p = 0.005, Figure 3). Females showed 53.7 million neurons/g and males 25.8 million neurons/g. Non-neuronal density was 72.2 million/g in women, and 40.8 million/g in men (p = 0.007), a significant difference of 43.5%.

### Ratio of non-neuronal/neuronal number

**Figure 4:**
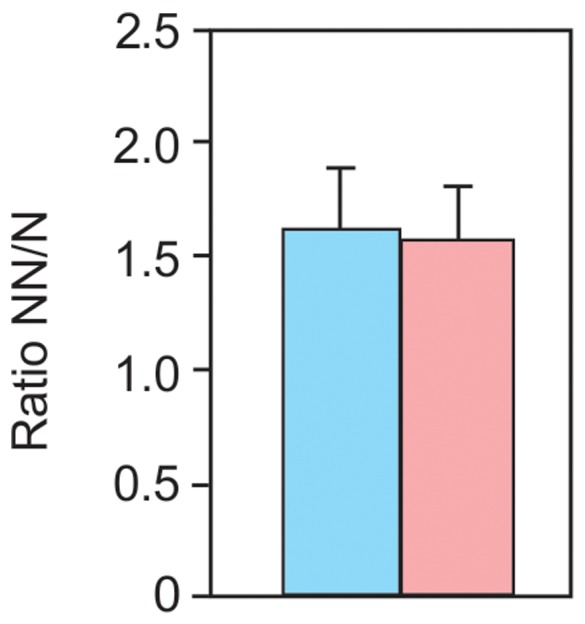
Ratio between non-neuronal and neuronal cells. No difference was found between men and women (p = 0.837). The bars indicate the mean for males (blue bar) and females (pink bar); error bar represents standard deviation.

The ratio between non-neuronal and neuronal cells found in human olfactory bulbs of women and men was similar (average for women was found to be 1.54±0.80 and for men 1.72±1.00; p = 0.786; see Figure 4). This value is very close to that found in other regions of the human brain when the isotropic fractionator method was utilized, such as the gray matter of the cerebral cortex with a ratio of 1.48, and the whole brain with a ratio of 1 [42], [43].

## Discussion

In the present study, we demonstrate that there is a pronounced sex-related difference in the absolute number of cells in the human olfactory bulb, both of neurons and non-neuronal cells, females having higher numbers than males.

### A general issue: the relation between brain cellularity and function

Can a larger number of neurons in female olfactory bulbs have a functional significance? To what extent can it be related to sexually dimorphic behavior?

The first question above requires an analysis about to what extent cellularity can be related to function in the nervous system in general. There are many arguments for this relation [51], [52]. A first line of thinking is evolutionary [53]. The number of cells, both neurons and glial cells, increase in each mammalian order as a function of brain size, and this increase follows predictable, order-specific, scaling equations [51], [52]. Most orders follow power equations, while primates follow a linear equation, an important difference that has optimized the increase of cell numbers across primates within larger, but more and more cell-dense brains. These scaling equations are true for the cerebral cortex, the cerebellum and the remaining regions as a whole. Generally speaking, larger brains with larger number of neurons in each order correlate with the functional complexity provided by these brains, as is the case of humans among primates. However, traditional indexes of evolutionary achievements such as body size, brain mass and volume, cerebral cortical mass, cortical folding index, and encephalization quotient, are not entirely satisfactory to explain the functional achievements of every species. A recent work [54] has highlighted very nicely this aspect, by studying the cellularity of an elephant, a mammal with a brain 3-fold larger than humans. The elephant brain, however, shows 1/3 less neurons in the cortex and 3 times more in the cerebellum. It is conceivable, thus, that the neuron-rich cerebellum takes care of the enormous body of the elephant, while the neuron-poor cortex is sufficient for the simple behavioral repertoire of the species.

Absolute cell numbers can therefore be more appropriately related to function in general, than other indexes based on volume of the brain and brain regions, either obtained by postmortem anatomy and histology, or by *in vivo* neuroimaging. However, it is obvious that other important parameters have to be considered, such as synapses and therefore circuits, not only because they define the information processing strategies of each brain region, but also because they provide the system with an important element for functional richness – plasticity [55]. However, quantitative methods to assess the absolute number of synapses and circuits are still not available, what hinders the use of these parameters for approaching the issue of sexual dimorphism in the human brain.

A second line of reasoning concerns the olfactory bulb specifically. It has been shown that interneurons therein are the most numerous among OB cells, and that they display an important characteristic: most of them are anaxonal cells that establish reciprocal synapses with projection neurons and among themselves [32]. This microstructural organization provides a distributive profile to the network, conveying to the neural hubs downstream a non-topographic, diffuse flow of olfactory information [33]. Given this diffuse nature of the information processed by the OB, it is conceivable that the larger the number of these neurons in the olfactory bulb, the more capable it becomes for processing more information to be analyzed by the piriform cortex, cortical amygdala, and other OB targets.

An indication for the relation between the larger number of OB neurons in women, and functional, sex-related differences, may emerge from studies that compare the ability of either sex to perceive and recognize odorants. Although men and women reportedly have approximately the same number of smell receptors [56], women's ability to perceive and identify odorants – functions attributed to the OB (see below) - is more accurate than men’s, considering olfactory tasks from simple threshold sensitivity to more complex episodic recognition and identification of odorants [10], [22]. Thus, chemosensory transduction is probably equal in both sexes, but processing of this information in the bulb becomes differentiated in women as compared to men. This improved ability of women is thought to imply an essential role of estrogen levels [15], [57], [58], and the targets of hormonal action should be looked for in the central nervous system.

Accordingly, evidence for better olfactory function in women has been produced in studies employing functional neuroimaging. In tasks where women and men identify and discriminate different types of odorants, females show a larger number of voxels activated in frontal and temporal lobes than men, for the same odorants [27]. Women’s superiority, however, was interpreted to be cognitive or emotional, rather than perceptual, since equal activation for both sexes was seen in the piriform and insular cortices [26].

### The olfactory bulb and sensitivity to odorants

The specific role of the OB in olfaction is not well established, particularly in humans. Based on the fact that it is the first synaptic stage after receptor transduction at the olfactory epithelium, most authors attribute simpler, discriminative functions to this region of the brain, namely odor threshold, discrimination, and identification [28], [33]. After chemosensory transduction, specifically coded odorant information is conveyed to a topographically characteristic set of glomeruli in the OB [33], but this topography is not maintained downstream in the olfactory nuclei, piriform cortex and cortical amygdala, among other targets. Due to the fact the OB volumes have not increased as much as other regions across species, another hypothesis has been offered, attributing navigational functions based on olfactory cues, to the OB [59]. Whether or not this hypothesis holds true for humans, is unknown.

One of the sex differences related more directly to the olfactory bulb refers to plain sensitivity to odorants. This includes threshold identification of each odorant, and odor discrimination. Some studies describe a higher sensitivity for particular odorants by women [58], [60], while others fail to show any discernible sex difference [10], [22]. According to Dalton and his collaborators [60], this sex-related difference in favor of women may be a result of repeated exposure to some specific odorants, a form of learning that would be more efficient in women than in men. The superiority of women in acquired olfactory sensitivity becomes more marked when the repeated exposure occurs during the reproductive phase [60]. To these authors, the results suggest that the olfactory-induction process may be associated with female reproductive behaviors such as pair bonding and kin recognition. In their experiments [60], women and men started with equal abilities to sense threshold olfactory and taste stimuli before puberty, but only postpubescent women showed an increase in sensitivity to some odorants such as benzaldehyde after repeated exposure. However, no difference was found to saccharin taste recognition, suggesting that this superior sensory ability of women is modality-specific and independent of familiarity [60]. Since no difference was found at the receptor level [56], any biological sex-related difference has to be searched downstream the olfactory epithelium, including the olfactory bulb.

In our study, we quantified the olfactory bulbs of females after the reproductive period (over 55 years old), and observed that the total numbers of neural cells, both neurons and non-neuronal cells, were higher in females when compared to males, what places the olfactory bulb as a possible site for a hardware specialization in women that could explain the superior olfactory sensitivity described above. Whether this higher number of computational units in the female olfactory bulbs is inborn or acquired during postnatal life under hormonal influence, remains to be determined. On the other hand, since female superiority is maintained after menopause, its biological determination should be under influences other than hormones. This seems to be the case of GABAergic receptors, that have been shown to determine sex-related differences of olfactory-driven reproductive behavior in rodents, and cause definitive quantitative differences in the number of the accessory olfactory bulb neurons [61], [62]. The accessory olfactory system as recognized in rodents and other animals, however, has not been proven to exist in humans [63].

### The role of reproductive hormones

Would reproductive hormones be the relevant factors for better identification of odors by women? There is evidence that estrogens increase while androgens depress olfactory performance [57]. Therefore, it would be conceivable that prepubescent subjects do not show sex differences in olfactory abilities, once reproductive hormones display equally low levels in both sexes during this period of life. However, this hypothesis was not confirmed, since prepubescent girls were shown to outperform boys for tasks involving odor identification [15], as much as older women (over 60 years old) in comparison with age-matched men, despite the decline in hormonal levels that both show after menopause and andropause [19]. Reproductive hormones and aging, therefore, do not seem to play a discernible role in the superior olfactory abilities of women. Our results are compatible with this conclusion, since women from our sample, older than 55 years, have 40% more cells in the olfactory bulb - neurons and non-neuronal cells - than men at the same age range.

Previous studies showed a correlation between OB volume and olfactory functions, since OB volumes decline with age together with a decrease of smell function [27]–[29]. MRI studies [28] in adult men and women (19 to 79 years old) showed that on average, left and right OB volumes of men are larger than women’s. For both, left and right OB volumes are stable up to the 4^th^ decade of life but decrease thereafter. Some of the functions tested showed significant correlation with OB volumes, but there was no functional dimorphism. In contrast, Gur and collaborators [64] showed that women (18 to 45 years old) have larger volumes of orbitofrontal cortex (BA 10, 11 and 25), a group of regions considered to be part of the olfactory system, and that reportedly provide modulatory feedback information to the mammalian olfactory bulb [65], [66]. In these regions, the concentration of gray matter was found to be higher in young women than in men [67]. According to this work, the measurements of gray matter concentration might reflect the organization of layers or the density of neurons. This imaging evidence concurs with our quantitative results, since we show that the female olfactory bulb has higher total cell density, as well as higher neuronal and non-neuronal cell densities than men, despite no significant difference in mass.

Although the olfactory bulb is reportedly larger in men, as per neuroimaging morphological criteria, adult women (20–44 years old) still have more voxels activated in right temporal lobe and left frontal lobe when both are exposed to the same odorants [27]. Additionally, women of different ages (18–83 years old) have larger olfactory evoked potential amplitudes to amyl acetate than men [23], and higher cerebral blood flow and cerebral metabolic rate of glucose utilization (18–33 years old) [30]. This may mean that female OBs transfer a greater amount of excitatory olfactory information to the subsequent cortical stages than is the case for males, what would agree with women’s richer neuronal machinery in their olfactory bulbs, as shown in this work.

### Does adult neurogenesis influence cellularity of the human olfactory bulb?

The OB is a region that undergoes neurogenesis in many mammalian species [32], [68]. According to Lledo and collaborators [69], environmental influences lead to changes in OB volume and to improvement of sensory abilities, what could be attributed to adult neurogenesis. Olfactory bulb neurogenesis is well documented in animals, but the extension to which it occurs in humans has remained controversial [70]. In a recent study, Bergmann and coworkers [35] quantified the number of new cells in the olfactory bulb by measuring ^14^C in the DNA of humans who were born during the 1950 s, when atomic bomb tests were performed at the earth’s surface. This technique allows establishing the birthdate of cells and their cycle. When they undergo mitosis, atmospheric ^14^C integrate into the DNA with the same concentration as that in the atmosphere, decaying afterwards according to a predictable equation that depends on the number of cell divisions after the period of isotope incorporation. Analysis of the ^14^C concentration in postmortem olfactory bulb DNA from adult humans revealed that only 2.0–3.4% non-neuronal cells and 0.008% neurons are added annually to the cell population of the olfactory bulb, the latter corresponding to <1% of new neurons in each 100 years. These figures contrast enormously with those measured in rats, which correspond to more than 50% new olfactory neurons per year [71]. The possibility that irradiation from the ^14^C atoms incorporated into the DNA could influence these experiments by blocking neurogenesis does not seem likely, since the same group succeeded in identifying neurogenesis in the dentate gyrus and in the striatum of a similar cohort of human subjects, using the same technique [72], [73]. Adult neurogenesis has also been demonstrated in the olfactory epithelium [74], but refuted to occur in the cerebral neocortex [75], although no specific focus on olfactory sectors of the cortex has been described.

Neurogenesis in rodents have been associated to functions as olfactory memory formation, social interactions and odor discrimination [69]. The lack of significant neurogenesis in the human olfactory bulb requires discarding the hypothesis that these functions are equally associated with this phenomenon in our species [35].

Based on the evidence that addition of new neurons is minimal after birth in the human olfactory bulb, we can safely conclude that our results are representative of the real number of neurons in the adult olfactory bulb, and strongly support the hypothesis that sex differences in cellularity therein derive either from embryonic proliferation and/or to postnatal cell death controlled by hormones or by inhibitory neurotransmitters.

### The influence of aging

Studies about aging in the human OB showed that women maintain a better performance to identify odors than men at all ages [19]. Peak performance occurs in both sexes around the fourth decade, and declines pronouncedly thereafter [28]. People over 70 years old have moderate loss of neurons and fibers [76].

Studies about age-related changes in olfactory function are conflicting (see [77] for a recent review). Maresh and collaborators [78] have not found significant differences with age. However, Meisami and others [63] observed a decrease in the number of glomeruli and mitral cells in the elderly. In accordance with this result, Sama-ul-Haq and collaborators [79] also showed a decrease in the number of mitral cells and diameter of their nuclei related to age in males and females. Other changes were related to aging as well, for example a decrease of 13% to 15% of olfactory bulb volume [57]; a decrease of 1% per year of olfactory fibers [19] and a reduction in the number and diameter of glomeruli of about 10% per decade [78]. Sex differences related to age in the olfactory bulb are also controversial. Sama-ul-Haq and collaborators [80] observed that males have a greater number and lower diameter of glomeruli than females between 20 and 39 years, while for ages older than 40, both number and diameter of glomeruli become lower in males than in females. However, in another study [78] no age-related sex difference was observed. Moreover, histological studies showed that there is an age-related loss of neurons in the olfactory bulb and a decrease in thickness of the glomerular layer, size and concentration of mitral cells per unit area [57], [79], [80]. Interestingly, old women showed less age-related decline in olfactory abilities when compared to old men at the same ages [81]. If decline of the olfactory abilities is related to a numerical decrease of neurons, it is possible that the decline in the number of neurons in women is less pronounced than in men. In the present study, we did not evaluate the loss of neuronal number because our sample was composed by postmortem bulbs with ages higher than 55 years old. In any case, despite the reported decline of some olfactory functions in the elderly [77], sexual dimorphism is maintained with aging.

## Conclusions

Our results demonstrate a pronounced sex-related difference in the absolute number of total, neuronal and non-neuronal cells, favoring women around 40%, even when corrected for mass. While in animals neuronal numbers in the OB could be influenced by adult neurogenesis, recent work has shown that this is not the case for humans. This large quantitative sexual dimorphism in the human olfactory bulb may be the morphological surrogate of sex differences in olfactory functions, most of which favor women as compared to men.
